# Family Vulnerability Scale: validity evidence in primary health care

**DOI:** 10.11606/s1518-8787.2023057005329

**Published:** 2024-04-01

**Authors:** Evelyn Lima de Souza, Flávio Rebustini, Ilana Eshriqui, Francisco Timbó de Paiva, Eliana Tiemi Masuda, Ricardo Macedo Lima, Daiana Bonfim

**Affiliations:** I Hospital Israelita Albert Einstein Centro de Estudos, Pesquisa e Práticas em APS e Redes São Paulo SP Brasil Hospital Israelita Albert Einstein. Centro de Estudos, Pesquisa e Práticas em APS e Redes. São Paulo, SP, Brasil; II Universidade de São Paulo Escola de Artes, Ciências e Humanidades Departamento de Gerontologia São Paulo SP Brasil Universidade de São Paulo. Escola de Artes, Ciências e Humanidades. Departamento de Gerontologia. São Paulo, SP, Brasil

**Keywords:** Health Vulnerability, Family Characteristics, Validation Study Primary Health Care, Population Health Management

## Abstract

**OBJECTIVE:**

Investigate evidence of validity of the Family Vulnerability Scale (EVFAM-BR) as an instrument to support population-based management in primary health care (PHC), in the scope of Health Care Planning (PAS).

**METHODS:**

This is a psychometric study to assess any additional evidence of the internal structure of EVFAM-BR using confirmatory factor analysis (CFA) and network analysis (NA). A preliminary version of the scale with 38 items was submitted to patients of PHC facilities that use the PAS methodology, distributed across the five regions of Brazil. For the primary CFA data, factor loadings and predictive power (R^2^) of the item were used. Seven model adjustment indices were adopted and reliability was measured by three indicators, using Bayesian estimation.

**RESULTS:**

The preliminary version of the scale was applied to 1,255 patients. Using the AFC, factor loadings ranged from 0.66 to 0.90 and R^2^ from 0.44 to 0.81. Both the primary indicators and the model adequacy indices presented satisfactory and consistent levels. According to the NA, the items were appropriately associated with their peers, respecting the established dimensions, thus demonstrating sustainability and stability of the proposed model.

**CONCLUSIONS:**

The evidence of validity presented by EVFAM-BR indicates, for the first time in Brazil, a concise instrument that is able to assertively measure family vulnerability, potentially supporting population-based management.

## INTRODUCTION

The health system organization, with a focus on strengthening primary health care (PHC) and as coordinator of care and organizer of the Health Care Network, is critical for the management of work processes and production of results in health^1^. It implies changing the predominant service management model in the Brazilian National Health System (SUS), which is based on the provision of services, into a population health management model, or population-based management, which recognizes the needs of the patient population, the context in which they are inserted, their social determinants of health, stratification by health risks to qualify the care provided, and the search for reducing health inequalities^2^.

In this sense, the Health Care Planning (PAS) methodology, proposed by the National Council of Health Secretaries^3,4^, is a strategy to organize work processes in PHC in order to promote population-based management. PAS is based on the discussion conducted by Mendes et al.^2^ about the organization of service provision, according to the demand profiles of the territory, and the Chronic Care Model^5^, which determines levels of care management according to risk stratification of subpopulations.

In addition to exposure to risks from the classic reasoning of epidemiology, in the health field, vulnerability has been discussed since the 1990s concerning social determinants of health as a set of factors that cause damage or condition of interest to the public health, being used as an indicator of social inequality, with increasing relevance based on studies of susceptible populations^6^. Considering the family context as one of the determinants of the health-disease process, families would have to be stratified by level of vulnerability in order to plan care and prioritize the most vulnerable ones. However, although the health care literature shows different definitions of family vulnerability, this concept is broad and difficult to measure^6^, involving multiple factors such as health status, income, and education of family members, as well family dynamics, among others^7,8^.

Some initiatives have proposed the development of instruments to measure family vulnerability, which can be used by PHC to plan care^10,11^. However, these initiatives have not advanced in validity, presenting limited use in Brazil, a continental-size country with different socioeconomic and cultural realities.

In PAS, a scale for stratifying family vulnerability has been developed and validated, so that it can be standardized nationwide in PHC^12^, in agreement with the organization process of population-based management. The Family Vulnerability Scale (EVFAM-BR)^12^ has 14 items divided into four dimensions (income, health care, family, and violence), answered with yes or no by a family member. Every positive answer to an item adds one point to the final score of the family vulnerability classification: low vulnerability (score of 0 to 4), moderate vulnerability (score of 5 to 6), and high vulnerability (score of 7 to 14).

A prior study^12^ described the EVFAM-BR development and validity stages using exploratory factor analysis (EFA). However, for validity of an instrument with enough robust evidence to support its recommendation, additional evidence must be investigated. Therefore, this study aims to investigate evidence of validity of the Family Vulnerability Scale as an instrument to support population-based management in PHC, in the PAS scope in Brazil.

## METHODS

### Study design

This is a psychometric study that seeks additional evidence of the internal structure of EVFAM-BR through confirmatory factor analysis (CFA) and network analysis (NA). The recommendations from the Standards for Educational and Psychological Testing, of the American Educational Research Association (AERA), the American Psychological Association (APA), and the National Council on Measurement in Education (NCME)^13^ to analyze sources of evidence were adopted. This study was approved by the Research Ethics Committee of Hospital Israelita Albert Einstein, report CEP 3.674.106, on October 22, 2019, CAAE 12395919.0.0000.0071.

### Study setting and population

A prior study described the development of the EVFAM-BR^12^ and presented evidence of its content validity and internal structure using an exploratory qualitative study with PHC health professionals. The 38-item scale version showed satisfactory evidence of content validity^12^ and was applied to patients of 11 basic health units (UBS): one in the North region (Roraima), one in the Northeast region (Pernambuco), two in the Central West region (Mato Grosso), five in the Southeast region (São Paulo and Minas Gerais), and two in the South region (Paraná).

The UBS selection criteria were: 1) units that adopt the PAS methodology; 2) selection of at least one UBS in each of the five Brazilian geographic regions; and 3) UBS located in the most populous municipalities with the largest population in the region.

Given the covid-19 pandemic, data collection was performed in two stages: the first between June and November 2020 through telephone contact with patients from UBS units located in São Paulo. The researchers obtained patient identification and contact information after consent from the respective UBS management. The second stage was conducted between May and August 2022 in remaining UBS units, through face-to-face interviews with patients who attended the respective services on data collection days. In both stages, the inclusion criterion was participants aged 18 years or older.

After the patients accepted the invitation to participate in the study and signed the informed consent form, the interviewers applied a structured questionnaire from the preliminary version of EVFAM-BR with a participant characterization questionnaire. RedCap^14^ software was used for data collection and storage.

### Statistical analysis

#### Confirmatory Factor Analysis

For the primary data of the CFA, factor loadings and the predictive power (R^2^) of the item were used. The model adjustment indices adopted were: χ^2^/df; non-normed fit index (NNFI ≥0 .95), comparative fit index (CFI ≥ 0.95), goodness fit index (GFI ≥ 0.95), Tucker-Lewis index (TLI), root mean square error of approximation (RMSEA ≤ 0.08), and root mean square of residuals (RMSR ≤0.8). The model tested in the AFC was the factorial solution found in the initial study of the EFA^12^.

Reliability was measured by three indicators: Cronbach’s alpha^15^, greatest lower bound or GLB^16^, and Omega^17^, using Bayesian estimation^18^.

#### Network analysis

Network analysis has been used in different settings and applications in the last decade, such as: assessment of symptoms^19^, psychological networks^20^, and post-traumatic stress^21^, and in the development of measurement instruments^22^. However, its use is incipient in studies on the development of measurement instruments in Brazil.

A network analysis usually has two steps: 1) estimation of a statistical data model, from which some parameters can be represented as a weighted network between assessed variables; and 2) analysis of the weighted network structure using measurements taken from graph theory^25^.

Our study used the high-dimensional undirected graph estimation (HUGE)^26^ technique as the estimator and the extended Bayesian information criteria (EBIC) as the criteria. HUGE uses two estimation procedures: the neighborhood search algorithm^27^ and the Lasso graph algorithm^28^. The graph nodes were positioned using the Fruchterman and Reingold algorithm^29^, which is based on the strength and connectivity between the nodes. Each node represents an item of the instrument.

Four indicators were adopted to evaluate the generated model: betweenness, which evaluates the efficiency with which a node connects to others; closeness, which assess how easy information reaches other nodes from a specific node; strength or degree, which shows how much a node is connected to the rest of the network^30^; and, finally, expected influence, which evaluates the nature and strength of the cumulative influence of a node within the network and, therefore, its expected role in activation, persistence, and remission^31^.

For both techniques (CFA and NA) a 5000 bootstrap was used. Analyses were performed in JASP 16.04 software. Absolute and relative frequencies, mean, standard deviation, and variation were used to characterize the participants.

## RESULTS

In total, 1,584 patients were invited to participate in the study; of these, 1,505 (95%) accepted it, and 1,255 completed the application of the preliminary version of the proposed scale. Mean age of participants was 43 years old (standard deviation: 15 years), most of them were female (43.9%), had brown skin (50.9%), 12 to 15 years of education (38.2%), received up to one minimum wage (26.1%), had no health insurance (83.7%), and were born in the Northeast region (34.0%), followed by the Southeast region (27.6 %), South (13.8%), North (11.2%), Central West (10.1%), and another country (1.7%).

**Figure 1:**
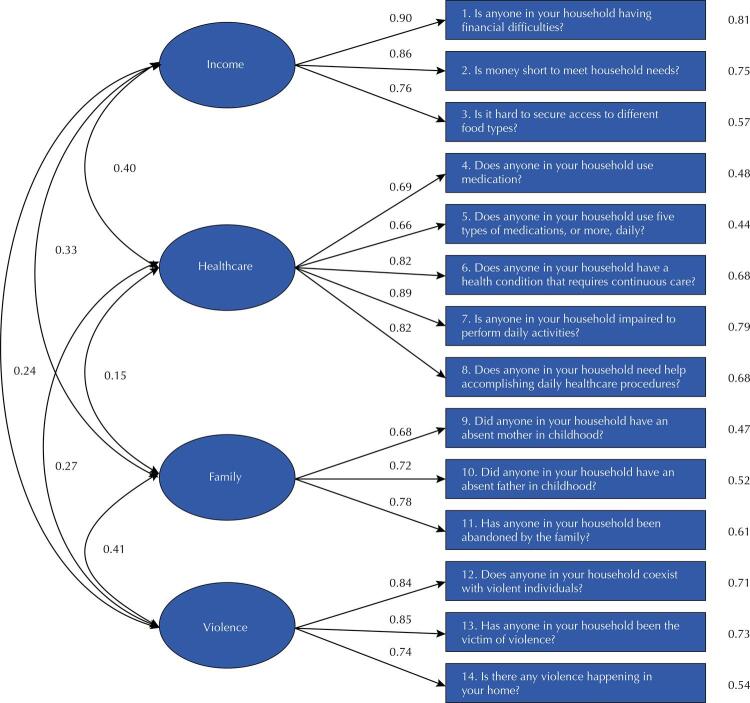
Path diagram.

Using the CFA, factor loadings ranged from 0.66 to 0.90 and the predictive capacity of the item (R^2^) from 0.44 to 0.81 (Figure 1). The “income” dimension presented factor loadings ranging from 0.75 to 0.90; the “health care” dimension from 0.66 to 0.89; the “family” dimension from 0.68 to 0.72; and the results of the “violence” dimension showed loadings between 0.74 and 0.85 – all of them at satisfactory levels. In addition to the primary indicators, the quality indices of the model were X^2^_(71)_ = 1.56, p = 0.0017; NNFI = 0.99, CFI = 0.99, GFI = 0.99, TLI = 0.99, RMSEA = 0.0218 (95%CI 0.0135–0.0293), and RMSR = 0.07. Covariance between factors ranged from 0.15 to 0.41.

Reliability indices with Bayesian estimation were Cronbach’s alpha = 0.70 (95%CI 0.67–0.75), McDonald’s omega = 0.71 (95%CI 0.68–0.73), and GLB = 0.83 (95%CI 0.81–0.84) – all of them at satisfactory levels.

This way, both the primary indicators and the model adequacy indices were at satisfactory and consistent levels.

**Figure 2:**
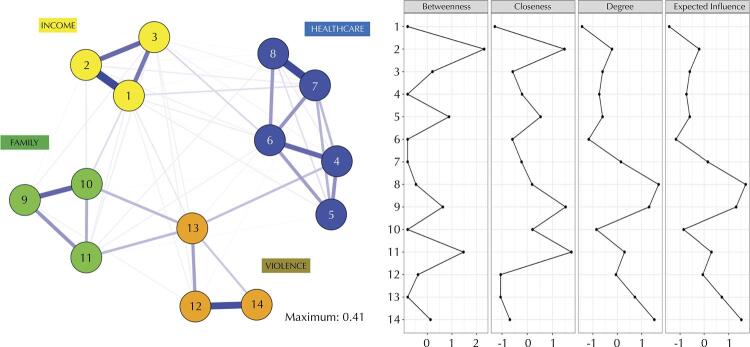
Network analysis (left) and Item Centrality Indices (right) of EVFAM-BR (z-score).

NA was applied to the previously developed model. Figure 2 shows the items were appropriately associated with their peers, respecting established dimensions. It again indicates sustainability and stability of the proposed model. Also, standardized centrality indices showed the roles of the items in the model.

For betweenness and closeness, item 2 “Is money short to meet household needs?” and item 11 “Has anyone in your household been abandoned by the family?” presented the best standardized values, indicating that both offer the best connection with the items of their dimension and those that favor the transfer of information between the nodes. For strength/degree, item 8 “Does anyone in your household need help accomplishing daily healthcare procedures?”, item 9 “Did anyone in your household have an absent mother in childhood?”, and item 14 “Is there any violence happening in your home?” These three items have the strongest connection in the network, with cumulative influence on the configuration of the model.

**Figure 3:**
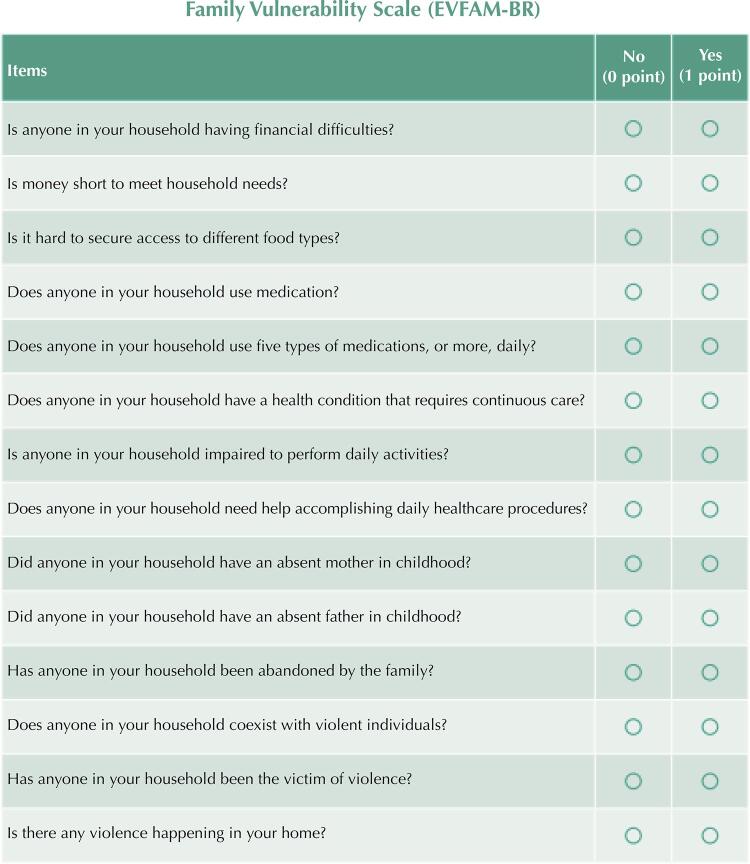
Family Vulnerability Scale (EVFAM-BR).

Source: Image extracted from the Roadmap for Using the Family Vulnerability Scale (EVFAM-BR) developed by the authors (available from: https://planificasus.com.br/arquivo-download.php?hash=48c3191b70c9ff22b8b336331fa417eaf87d366d&t=1692220483&type=biblioteca).

Figure 3 shows the final version of EVFAM-BR obtained from the evidence found in this study.

## DISCUSSION

The results of this study confirm the EVFAM-BR is a consistent and reliable model and reinforce the contribution of the information obtained with a combination of techniques to the development of an instrument that measures family vulnerability.

The adoption of multiple tests to adjust a model (instrument) met contemporary recommendations for validity evidence^13^, which has highlighted and recommended the need and contribution of models tested using multiple techniques. This combination seeks to improve instrument precision and quality by adding much more information to the model^32,33^. It also helps determine the best model when many potential solutions are available^34^. In addition, our study highlights the option of using the same sample in the exploratory factor analysis from the previous study^12^ and in the analyses presented here, understanding the techniques analyze data in a different and complementary way, and ensuring consistency of the final model of the instrument. In this sense, the literature shows that using more than one technique with the same database provides more information about the parameters and the functioning of the models^32,33^. In this context, the EVFAM-BR is a concise instrument with consistent evidence of validity.

The need for a validated scale that would allow the measurement of family vulnerability in different Brazilian scenarios appeared in the context of organizing work processes in PHC through the PAS, such as population registration, identification of subpopulations at risk, and stratification of family risk^4^. The organization of these aspects is critical considering that it is necessary to learn about the population and identify groups of health vulnerability in order to plan care using a population-based management model. Based on the PAS theoretical-methodological framework, which consists of implementing the Chronic Care Model^2^ to support the organization of PHC work processes, the development of a scale that qualifies the process to prioritize needs and plan care using a population-based management model contributes to this objective.

In essence, the scale was designed as a working instrument for community health agents (CHAs), who are Family Health Strategy (FHS) professionals recognized for their experience with the territory and connection with the enrolled families. In this perspective, it is easier for CHAs to communicate and identify problems in family dynamics, representing one of the most important channels for population-service communication^35^. In this context, EVFAM-BR is a tool with potential applicability by CHAs during home visits (as interviewers, since the questions are answered by a family member). The scale application can be appropriate at the time of family registration, qualifying the information obtained for population-based management. However, a wide range of professionals from PHC teams can measure family vulnerability using various tools (paper, application, electronic form, among others), settings (service visit, home visit, others) or even the self-application by the patient (for example, by text application on a smartphone). Of note, the information derived from the scale can contribute to the decision-making process in different areas of care.

EVFAM-BR is a tool that can support the work dynamics in PHC, providing opportunities for collaborative practice and comprehensive and equitable care, which helps overcome some challenges foreseen in the Sustainable Development Goals (SDGs) issued by the World Health Organization, in particular SDG 3 (Health and well-being), ensuring equitable access to quality health; and SDG 10 (Reduction of inequalities), promoting equal opportunities and reducing inequalities in health outcomes^36^.

The scale covers dimensions of social importance, such as income, health care, family, and violence^12^. Then, the interpretation of family vulnerability strata foreseen in EVFAM-BR^12^ can be incorporated into different actions and activities in PHC, enriching dynamic maps of the territory, organization of social and community actions, team discussions and planning of services that meet the needs of the population at local, regional, and national levels^10,37,38^. Considering the dynamics of the PHC territory and the possibility of periodic and systematic updating of the EVFAM-BR, its use can be helpful for monitoring the distribution of high and moderate vulnerability families in the territory, contributing to the assessment of adequacy and potential redistribution required in the territory.

Although the EVFAM-BR information is not included in health information systems, which would speed up the stratification process of enrolled families^10^, the scale items help identify other aspects of the family nucleus that impact the demand for health care beside those traditionally observed by teams and added to electronic records, such as the presence of a chronic condition, acute events, age group, sex, among others^39^.

## CONCLUSION

The territory vision by family vulnerability strata and care planning focused on identified needs are within the scope of the organization of PHC processes for population-based management, as recommended by PAS. In this context, the robust validity evidence presented by EVFAM-BR covering the national context constitutes a concise instrument that can measure family vulnerability with potential broad application by professionals in Brazil.
