# Supplementary material for: Family Vulnerability Scale: validity evidence in primary health care
Source: Rev Saude Publica. 2024 Apr 1;57(Suppl 3):5s. doi: 10.11606/s1518-8787.2023057005329 (PMC11037913; doi:10.11606/s1518-8787.2023057005329)
Supplement: Translation

# Translation: Escala de Vulnerabilidade Familiar: evidências de validade na atenção primária à saúde

**Authors:** Evelyn Lima de Souza, Flávio Rebustini, Ilana Eshriqui, Francisco Timbó de Paiva, Eliana Tiemi Masuda, Ricardo Macedo Lima, Daiana Bonfim

**Affiliations:** I Brasil Hospital Israelita Albert Einstein. Centro de Estudos, Pesquisa e Práticas em APS e Redes. São Paulo, SP, Brasil; II Brasil Universidade de São Paulo. Escola de Artes, Ciências e Humanidades. Departamento de Gerontologia. São Paulo, SP, Brasil

**Keywords:** Vulnerabilidade em Saúde, Características da Família, Estudo de Validação, Atenção Primária à Saúde, Gestão da Saúde da População

## Abstract

**OBJETIVO:**

Investigar evidências de validade da Escala de Vulnerabilidade Familiar (EVFAM-BR) como instrumento de apoio à gestão de base populacional na atenção primária à saúde (APS), no escopo da Planificação da Atenção à Saúde (PAS).

**MÉTODOS:**

Estudo de natureza psicométrica de evidências adicionais da estrutura interna da EVFAM-BR, por meio da análise fatorial confirmatória (AFC) e análise de redes (*network analysis*, NA). Versão preliminar da escala com 38 itens foi submetida a usuários de unidades de APS, que executam a metodologia da PAS, distribuídas nas cinco regiões brasileiras. Para os dados primários da AFC, utilizou-se as cargas fatoriais e o poder preditivo do item (R^2^). Foram adotados sete índices de ajustamento do modelo e a confiabilidade foi aferida por três indicadores, por meio de estimação bayesiana.

**RESULTADOS:**

A versão preliminar da escala foi aplicada a 1.255 usuários. Pela AFC, as cargas fatoriais variaram de 0,66 a 0,90 e R^2^ de 0,44 a 0,81. Tanto os indicadores primários como os índices de adequação do modelo estabeleceram-se em níveis satisfatórios e consistentes. A NA apontou que os itens se associaram de forma adequada com seus pares, respeitando as dimensões estabelecidas, o que indica, novamente, a sustentabilidade e estabilidade do modelo proposto.

**CONCLUSÕES:**

As evidências de validade apresentadas pela EVFAM-BR enunciam, de forma inédita no Brasil, um instrumento conciso, capaz de mensurar assertivamente a vulnerabilidade familiar, potencialmente apoiando a gestão de base populacional.

## INTRODUÇÃO

A organização do sistema de saúde, com foco no fortalecimento da atenção primária à saúde (APS) como coordenadora do cuidado e ordenadora da Rede de Atenção à Saúde (RAS), é elemento fundamental para a organização dos processos de trabalho e produção de resultados em saúde^1^. Isto implica transformar o modelo da gestão da oferta – predominante no Sistema Único de Saúde (SUS), pautado na oferta de serviços – no modelo da gestão da saúde da população, ou de base populacional, ou seja, reconhecer as necessidades da população usuária, o contexto em que vivem, seus determinantes sociais da saúde, a estratificação por riscos sanitários para qualificação do cuidado ofertado centrado nelas e a busca pela redução das desigualdades em saúde^2^.

Neste sentido, a metodologia da Planificação da Atenção à Saúde (PAS), proposta pelo Conselho Nacional de Secretários de Saúde^3,4^, conforma-se como estratégia para a organização dos processos de trabalho na APS, a fim de promover a gestão de base populacional. A PAS se baseia na discussão realizada por Mendes et al.^2^ sobre a organização da oferta de serviços, segundo os perfis de demanda do território, e no Modelo de Atenção às Condições Crônicas^5^, que determina níveis de gestão do cuidado segundo a estratificação de riscos das subpopulações.

Para além da exposição a riscos do raciocínio clássico da epidemiologia, no campo da saúde a vulnerabilidade tem sido discutida desde a década de 90, no âmbito dos determinantes sociais e de saúde, como um conjunto de fatores que causam qualquer dano ou condição de interesse à saúde pública, sendo utilizada como um indicador de desigualdade social e de crescente relevância a partir de estudos de populações suscetíveis^6^. Considerando o contexto familiar como um dos determinantes do processo saúde-doença, seria necessário estratificar as famílias por nível de vulnerabilidade, para programar o cuidado priorizando as mais vulneráveis. No entanto, embora a literatura em saúde apresente diferentes definições para o termo, o conceito de vulnerabilidade familiar é amplo e dificilmente mensurável^6^, relacionado a múltiplos fatores, como condição de saúde dos membros, renda, educação, dinâmica familiar, entre outros^7,8^.

Algumas iniciativas propuseram o desenvolvimento de instrumentos para mensuração de vulnerabilidade das famílias, que podem ser utilizados pela APS para o planejamento da assistência^10,11^. Porém, eles carecem de avanços nas evidências relacionadas a sua validação, apresentando limitações para utilização em um país como o Brasil, com dimensões continentais e múltiplas realidades socioeconômicas e culturais.

No âmbito da PAS, uma escala para estratificação da vulnerabilidade familiar tem sido desenvolvida e validada, para que possa ser utilizada de forma padronizada em âmbito nacional na APS^12^, em consonância com o processo de organização da gestão de base populacional. A Escala de Vulnerabilidade Familiar (EVFAM-BR)^12^ é composta por 14 itens, distribuídos em quatro dimensões (renda, cuidado em saúde, família e violência), respondidos de forma dicotômica (sim ou não) e autorreferida por um membro da família. Cada resposta positiva a um item contabiliza um ponto para o escore final da classificação da vulnerabilidade familiar: baixa vulnerabilidade (0 a 4 pontos), moderada vulnerabilidade (5 a 6 pontos) e alta vulnerabilidade (7 a 14 pontos).

Em estudo prévio^12^, foram descritas as etapas de elaboração e validação da EVFAM-BR por meio da análise fatorial exploratória (AFE). No entanto, no que diz respeito às etapas de validação de um instrumento com evidências robustas para a sua recomendação, há de se investigar evidências adicionais. Desta forma, este estudo tem como objetivo investigar evidências de validade da Escala de Vulnerabilidade Familiar como instrumento de apoio à organização da gestão de base populacional da APS, no escopo da PAS no Brasil.

## MÉTODOS

### Delineamento

Trata-se de um estudo de natureza psicométrica, na busca de evidências adicionais de estrutura interna da EVFAM-BR, por meio da análise fatorial confirmatória (AFC) e análise de redes (*network analysis*, NA). Adotou-se as recomendações para fontes de evidências estabelecidas no *Standards for Educational e Psychological Testing*, da Associação Americana de Pesquisa Educacional (Aera), da Associação Americana de Psicologia (APA) e do Conselho Nacional sobre Medicina Educacional (NCME)^13^. O estudo foi aprovado pelo Comitê de Ética em Pesquisa do Hospital Israelita Albert Einstein, parecer CEP 3.674.106, em 22 de outubro de 2019, CAAE 12395919.0.0000.0071.

### Cenário e População do Estudo

Estudo prévio descreveu a construção e apresentou evidências de validade de conteúdo e estrutura interna da EVFAM-BR^12^, por meio de estudo qualitativo exploratório com profissionais de saúde da APS. A versão da escala com 38 itens, que apresentou evidências de validade de conteúdo satisfatórias^12^, foi aplicada a usuários de 11 unidades básicas de saúde (UBS), sendo uma na região Norte (Roraima), uma na região Nordeste (Pernambuco), duas na região Centro-Oeste (Mato Grosso), cinco na região Sudeste (São Paulo e Minas Gerais) e duas na região Sul (Paraná).

Foram considerados como critérios para escolha das UBS: 1) unidades que adotam a metodologia da PAS; 2) seleção de ao menos uma UBS em cada uma das cinco regiões geográficas brasileiras; e 3) localização em municípios com maior população.

Frente ao cenário pandêmico da covid-19, a coleta de dados foi realizada em duas etapas, sendo a primeira entre junho e novembro de 2020, por meio de contato telefônico com os usuários das UBS localizadas em São Paulo. Neste contexto, os pesquisadores obtiveram informações de identificação e contato dos usuários após anuência das gerências dos respectivos serviços.

A segunda etapa ocorreu entre maio e agosto de 2022, nas demais UBS, com entrevistas presenciais com usuários que compareceram aos respectivos serviços nos dias de coleta de dados. Em ambas as etapas foi adotado como critério de inclusão o participante ter 18 ou mais anos de idade. Mediante o aceite do convite para participar da pesquisa e após a apresentação do termo de consentimento livre e esclarecido, os entrevistadores aplicaram um questionário estruturado da versão preliminar da EVFAM-BR, junto à caracterização do participante. O *software* RedCap®^14^ foi utilizado para coleta e armazenamento de dados.

### Análise Estatística

#### Análise Fatorial Confirmatória

Para os dados primários da AFC, utilizou-se as cargas fatoriais e o poder preditivo do item (R^2^). Os índices de ajustamento do modelo adotados foram: χ^2^/df; *Non-Normed Fit Index* (NNFI ≥ 0,95); *Comparative Fit Index* (CFI ≥ 0,95); *Goodness Fit Index* (GFI ≥ 0,95); Tucker-Lewis *Index* (TLI), *Root Mean Square Error of Approximation* (RMSEA ≤ 0,08) e *Root Mean Square of Residuals* (RMSR ≤ 0,8). O modelo testado na AFC foi a solução fatorial encontrada no estudo inicial da AFE^12^.

A confiabilidade foi aferida por três indicadores: alpha de Cronbach^15^, *Greatest Lower Bound* – GLB^16^ e Ômega^17^, por meio de estimação bayesiana^18^.

#### Análise de Redes

A análise de redes tem sido utilizada para diversos cenários e aplicações na última década, tais como: avaliação de sintomas^19^, redes psicológicas^20^ e estresse pós-traumático^21^, bem como no desenvolvimento de instrumentos de medida^22^. Contudo, sua aplicação nos estudos de desenvolvimento de instrumentos de medida no Brasil é incipiente.

Usualmente, a análise de redes é composta por dois estágios: 1) estimar um modelo estatístico de dados, a partir do qual alguns parâmetros podem ser representados como uma rede ponderada entre as variáveis observadas; e 2) analisar a estrutura da rede ponderada usando medidas tiradas da teoria dos grafos^25^.

Nosso estudo utilizou como estimador a técnica *High-dimensional Undirected Graph Estimation* (Huge)^26^ e, como critério, o *Extended Bayesian Information Criterio* (Ebic). A Huge trabalha com dois procedimentos de estimação: o algoritmo de busca em vizinhança^27^ e o algoritmo gráfico de Lasso^28^. Os nodos dos gráficos foram posicionados utilizando o algoritmo de Fruchterman e Reingold^29^, que é baseado na força e conectividade entre os nodos. Cada nodo representa um item do instrumento.

Para avaliar o modelo gerado, foram adotados quatro indicadores: intermediação (*betweeness*), que avalia a eficiência com que um nó se conecta a outros; proximidade (*closeness*), que avalia a facilidade com que a informação atinge os outros nós a partir de um nó específico; força (*strength or degree*), que representa o quanto um nó está conectado ao resto da rede^30^; e, por fim, a influência esperada (*expected influence*), que visa avaliar a natureza e a força da influência cumulativa de um nó dentro da rede e, portanto, o papel que se pode esperar que ele desempenhe na sua ativação, persistência e remissão^31^.

Para ambas as técnicas (AFC e NA) foi aplicada uma *bootstrap* de 5000. As análises foram efetuadas com o software JASP 16.04. Frequências absoluta e relativa, média, desvio-padrão e variação foram utilizados para caracterização dos participantes.

## RESULTADOS

Foram convidados a participar do estudo 1.584 usuários, dos quais 1.505 (95%) aceitaram e 1.255 completaram a aplicação da versão preliminar da escala proposta. Os participantes apresentaram média de 43 anos de idade (desvio-padrão de 15 anos), a maioria do sexo feminino (43,9%), da raça/cor parda (50,9%), com 12 a 15 anos de estudo (38,2%), recebia até um salário-mínimo (26,1%), não tinha plano de saúde (83,7%) e nasceu na região Nordeste (34,0%), seguido da região Sudeste (27,6%), Sul (13,8%), Norte (11,2%), Centro-Oeste (10,1%) e em outro país (1,7%).

Pela AFC, as cargas fatoriais variaram de 0,66 a 0,90 e a capacidade preditiva do item (R^2^) de 0,44 a 0,81 (Figura 1). A dimensão “renda” teve cargas fatoriais oscilando entre 0,75 e 0,90; a dimensão “cuidado em saúde” de 0,66 e 0,89; a dimensão “família” entre 0,68 e 0,72; e os resultados da dimensão “violência” tiveram cargas entre 0,74 e 0,85. Todos em níveis satisfatórios. Além dos indicadores primários, os índices de qualidade do modelo estabeleceram-se em: X^2^_(71)_ = 1,56, p = 0,0017; NNFI = 0,99, CFI = 0,99, GFI = 0,99, TLI = 0,99, RMSEA = 0,0218 (IC95% 0,0135–0,0293) e RMSR = 0,07. As covariâncias entre os fatores variaram entre 0,15 e 0,41.

**Figura 1:** Diagrama de caminhos.

Os índices de confiabilidade com estimação bayesiana foram de alpha de Cronbach = 0,70 (IC95% 0,67–0,75), ômega de McDonald = 0,71 (IC95% 0,68–0,73) e GLB = 0,83 (IC95% 0,81–0,84). Todos os indicadores em níveis adequados.

Desta forma, tanto os indicadores primários como os índices de adequação do modelo estabeleceram-se em níveis satisfatórios e consistentes.

A NA foi aplicada ao modelo estabelecido anteriormente. A Figura 2 demonstra que os itens se associaram de forma adequada com seus pares respeitando as dimensões estabelecidas. O que indica, novamente, a sustentabilidade e estabilidade do modelo proposto. Além disso, os índices de centralidade normatizados mostraram os papéis dos itens no modelo.

**Figura 2:** *Network analysis* (esquerda) e Índices de Centralidade dos Itens (direita) da EVFAM-BR (*z-score*).

Para a intermediação (*betweeness*) e para a proximidade (*closeness*), os itens 2 “Falta dinheiro para atender as necessidades do domicílio?” e 11 “Algum familiar já esteve em situação de abandono pela família?” apresentaram os melhores valores normatizados, indicando que ambos são os que melhor se conectam com os itens da sua dimensão e os que favorecem a passagem da informação entre os nós. Para a força/grau (*strenght/degree*), os itens 8 “Alguém no domicílio necessita de ajuda para realizar seus cuidados diários de saúde?”, 9 “Alguém no domicílio teve a mãe ausente durante a infância?” e 14 “Acontece violência em sua casa?”. Esse resultado indica que estes três itens são os que apresentam a maior força da conexão na rede e a influência cumulativa deles para a configuração do modelo.

A versão final da EVFAM-BR, obtida a partir das evidências apresentadas, encontra-se na Figura 3.

**Figura 3:** Escala de Vulnerabilidade Familiar (EVFAM-BR).

Fonte: imagem extraída do Roteiro para Utilização da Escala de Vulnerabilidade Familiar (EVFAM-BR), de elaboração dos autores (disponível em: https://planificasus.com.br/arquivo-download.php?hash=48c3191b70c9ff22b8b336331fa417eaf87d366d&t=1692220483&type=biblioteca).

## DISCUSSÃO

Os resultados deste estudo confirmam um modelo consistente e confiável da EVFAM-BR e reforçam a contribuição das informações obtidas com a combinação entre técnicas para o desenvolvimento do instrumento de mensuração da vulnerabilidade familiar.

A adoção de múltiplas testagens para o ajuste de um modelo (instrumento) atendeu as recomendações contemporâneas de evidências de validade^13^, que tem reforçado e recomendado a necessidade e a contribuição de modelos testados em múltiplas técnicas. Entende-se que essa combinação busca melhorar a precisão e a qualidade dos instrumentos, além de trazer muito mais informações ao modelo^32,33^, além de auxiliar na determinação de qual é o melhor modelo, quando há mais de uma possibilidade de solução^34^. Além disso, destacamos a opção de utilizar a mesma amostra para a análise fatorial exploratória no estudo prévio^12^ e nas análises aqui apresentadas, compreendendo-se que as técnicas analisam os dados de forma diferente e complementar, garantindo consistência do modelo final do instrumento. Neste sentido, a literatura aponta que a aplicação de mais de uma técnica sobre o mesmo banco contribui com mais informações sobre os parâmetros e no funcionamento dos modelos^32,33^. Neste contexto, a EVFAM-BR apresenta-se como um instrumento conciso e com evidências de validade consistentes.

A necessidade de uma escala validada que permitisse a mensuração da vulnerabilidade familiar nos diferentes cenários brasileiros surgiu no contexto de organização de processos de trabalho na APS, por meio da PAS, como o cadastro populacional, identificação das subpopulações de risco e estratificação de risco das famílias^4^. A organização destes torna-se fundamental a partir da concepção de que é preciso conhecer a população e identificar grupos de vulnerabilidade em saúde para, assim, programar o cuidado a partir da gestão de base populacional. Com base no referencial teórico-metodológico da PAS, que consiste em implantar o Modelo de Atenção às Condições Crônicas^2^ para apoiar a organização dos processos de trabalho da APS, a elaboração de uma escala que qualifique o processo de priorização das necessidades e programação do cuidado com gestão de base populacional contribui com este objetivo.

Em sua essência, a escala foi pensada como um instrumento de trabalho do agente comunitário de saúde (ACS), profissional da Estratégia Saúde da Família (ESF) reconhecido pela experiência com o território e vínculo com as famílias adscritas. Nesta perspectiva, o ACS apresenta maior facilidade em dialogar e identificar problemas na dinâmica familiar, conformando-se como uma das mais importantes vias de comunicação população-serviço^35^. Neste contexto, a EVFAM-BR apresenta-se como uma ferramenta com potencial de aplicabilidade pelo ACS durante as visitas domiciliares (no papel de entrevistador, uma vez que as questões são autorreferidas por um membro da família). Destaca-se que a aplicação da escala pode ser oportuna no momento do cadastro familiar, qualificando a obtenção de informações para a gestão de base populacional. No entanto, há possibilidades da mensuração da vulnerabilidade familiar por ampla gama de profissionais das equipes da APS, por meio de diversas ferramentas (papel, aplicativo, formulário eletrônico, dentre outros), contextos (consulta, visita, outros) ou, ainda, a autoaplicação pelo usuário (por exemplo, por aplicativo de texto no celular). Ressalta-se que as informações derivadas da escala podem contribuir para a tomada de decisão de diferentes esferas do cuidado.

A EVFAM-BR configura-se como ferramenta com potencial para apoiar a dinâmica de trabalho na APS, oportunizando a prática colaborativa, cuidado integral e equitativo, e, assim, favorecendo a superação de alguns dos desafios previstos nos Objetivos de Desenvolvimento Sustentável da Organização Mundial da Saúde, especificamente o objetivo 3 (Saúde e bem-estar), por meio da garantia do acesso à saúde de forma equânime e com qualidade; e o objetivo 10 (Redução das desigualdades), promovendo a igualdade de oportunidades e reduzindo as desigualdades de resultados em saúde^36^.

A escala abrange dimensões de importância social, como renda, cuidado em saúde, família e violência^12^. A partir disso, a interpretação dos estratos de vulnerabilidade das famílias previstos na EVFAM-BR^12^ pode ser incorporada nas mais diversas ações e atividades na APS, enriquecendo mapas dinâmicos do território, organização das ações sociais e comunitárias, discussões de caso em equipe e planejamento dos serviços da unidade que respondam às necessidades da população, em um contexto local, regional e nacional^10,37,38^. Considerando a dinamicidade do território da APS e a possibilidade de atualização periódica e sistemática da EVFAM-BR, sua utilização pode ser útil para o monitoramento da distribuição de famílias de alta e moderada vulnerabilidade no território, contribuindo para avaliação da adequação e possíveis necessidades de redistribuição do território.

Apesar das informações da EVFAM-BR não estarem contempladas nos sistemas de informação em saúde, o que agilizaria o processo de estratificação das famílias adscritas^10^, os itens da escala oportunizam a identificação de outros fatores do núcleo familiar, que impactam a demanda em saúde para além daqueles tradicionalmente observados pelas equipes e registrados em prontuário eletrônico, como a presença de condição crônica, eventos agudos, faixa etária, sexo, entre outros^39^.

## CONCLUSÃO

A visão do território por estratos de vulnerabilidade das famílias e a orientação da programação do cuidado centrada nas necessidades identificadas estão no escopo da organização de processos da APS para a gestão de base populacional, como preconizado pela PAS. Nesta conjuntura, as evidências de validade apresentadas pela EVFAM-BR enunciam, com evidências de validade robustas e que contemplam o cenário nacional, um instrumento conciso capaz de mensurar a vulnerabilidade familiar com potencial de ampla aplicação por profissionais no Brasil.
